# Do Giant Sequoias Regenerate in Large Crown Fire Patches?

**DOI:** 10.1002/ece3.73306

**Published:** 2026-03-27

**Authors:** Chad T. Hanson, Tonja Y. Chi, Maya Khosla, Craig Swolgaard

**Affiliations:** ^1^ Earth Island Institute Berkeley California USA; ^2^ Wildlife Ecologist South Lake Tahoe California USA; ^3^ Wildlife Ecologist Rohnert Park California USA; ^4^ Wildlife Ecologist Georgetown California USA

**Keywords:** forests, giant sequoia, high severity, Sierra Nevada, wildfire

## Abstract

Research indicates that the giant sequoia, a serotinous conifer and the world's most massive tree species, is positively associated with high‐severity fire for effective reproduction. However, land managers hypothesize that portions of giant sequoia groves could be lost in large crown fire areas due to a lack of regeneration, extensive montane chaparral cover, and long distances to the nearest surviving sequoia seed trees. Based on these hypotheses, rollbacks of environmental laws to facilitate intensive logging and tree plantation establishment are now proposed in giant sequoia groves on national forests and national parks, in the name of wildfire management and reforestation. Yet existing research is sparse, particularly regarding postfire sequoia regeneration that reaches the size of small trees (≥ 140 cm tall), which are most likely to survive to maturity. We investigated this issue in 62 field plots within the largest high‐severity fire patches in Redwood Mountain Grove, Sequoia, and Kings Canyon National Parks, four years post‐fire. These patches are dominated by crown fire but also include areas of high‐intensity surface fire. At four years post‐fire, we found sequoia regeneration density (mean = 19,478/ha) that was more than 21 times higher than initial modeling projected. Within the high‐severity fire category, we found no correlation between fire severity or percent montane chaparral cover and giant sequoia small tree density, but found the percentage of all sequoia regeneration comprised by small trees is significantly higher in crown fire areas. The mean distance to the nearest live sequoia is now significantly shorter than reported at one year post‐fire, suggesting that some live sequoias were not recognized as still living in initial evaluations. Our findings indicate that giant sequoia regeneration is thriving in large high‐severity fire areas dominated by crown fire.

## Graphical abstract

While it is widely recognized that giant sequoias are serotinous and reproduction is positively associated with high‐severity fire, land managers hypothesize that giant sequoias cannot effectively reproduce in large crown fire patches. We investigated this issue in two large high‐severity fire patches, dominated primarily by crown fire, in Redwood Mountain Giant Sequoia Grove. We found abundant sequoia regeneration in crown fire areas, with a mean of nearly 20,000/ha.

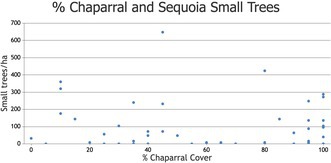

## 1 Introduction

The giant sequoia ( *Sequoiadendron giganteum* ), the world's most massive tree species, is a serotinous conifer (Harvey et al. 1980; Weatherspoon 1990; Harvey and Shellhammer 1991) for which seed release is almost exclusively triggered by fire. This evolutionary trait originated approximately 120–65 million years ago for modern conifer species, and much earlier for ancestor species (He et al. 2016; Lamont et al. 2019, 2020). Similar to other serotinous conifers globally (Schoennagel et al. 2003; Briand et al. 2015; Su et al. 2015; Ladd et al. 2022; Pelletier and de Lafontaine 2023; Liu et al. 2025), it has been found that giant sequoias are strongly associated with high‐severity fire for effective reproduction (Stephenson 1994; Meyer and Safford 2011; Hanson, Chi, Khosla, et al. 2024; Hanson, Chi, Baker, et al. 2024). High‐severity fire includes both high‐intensity surface fire, where foliage and trees are killed by radiant heat, and crown fire, where flames reach the upper canopy and consume foliage (Stephenson, Caprio, et al. 2024; Stephenson, Soderberg, et al. 2024). The former is defined by RdNBR (Relativized Differenced Normalized Burn Ratio) fire severity values 641–800, while the latter (crown fire), at the upper end of the high‐severity fire spectrum, is defined by RdNBR values > 800 (Soderberg et al. 2024; Stephenson, Caprio, et al. 2024; Stephenson, Soderberg, et al. 2024). Large high‐severity fire patches include a mix of these effects.

However, at present there is no scientific consensus regarding giant sequoia regeneration *within* the high‐severity fire category. Some research has found substantial giant sequoia regeneration in crown fire areas, but is either limited to less than two years post‐fire (Hanson, Chi, Khosla, et al. 2024; Meyer et al. 2024; Soderberg et al. 2024), or to a small study site (Hanson, Chi, Baker, et al. 2024). Land management agencies hypothesize that giant sequoias are adapted to small patches of high‐intensity surface fire but are not resilient to crown fires (USDA 2022; USDOI 2023). One hypothesis suggests that in crown fire areas within high‐severity fire patches, the peduncles holding cones to branches could be incinerated, causing cones to fall into intense flames where cones and seeds would be consumed, inhibiting reproduction (Stephenson, Caprio, et al. 2024; Stephenson, Soderberg, et al. 2024; Keeley and Pausas 2025), but the idea has not been investigated. Based on this hypothesis, along with the hypotheses that high levels of montane chaparral cover and very long distances to surviving seed trees in larger crown fire patches could prevent giant sequoia reproduction and succession, federal agencies have expressed concern that portions of giant sequoia groves could be lost due to crown fire. The agencies have suggested that such an outcome might transpire either because no sequoia regeneration would occur, or sequoia seedlings that were initially evident at one or two years post‐fire would all quickly die (USDA 2022; USDOI 2023; Soderberg et al. 2024; Stephenson, Caprio, et al. 2024; Stephenson, Soderberg, et al. 2024).

Driven by these hypotheses and the significant mortality of large giant sequoias from recent large fires, a widespread program of mechanical thinning and other logging in giant sequoia groves is now being implemented to curb wildfires and prevent high‐severity fire (Shive et al. 2022; USDA 2022). Legislation is now proposed in the U.S. Congress that would override the Endangered Species Act, the National Environmental Policy Act, and the Wilderness Act to allow any form of logging in giant sequoia groves in national forests and national parks, including in Wilderness Areas, for the stated goal of preventing high‐severity fires in the sequoia groves.

Conversely, a growing body of science indicates that mechanical thinning and postfire logging may not effectively reduce wildfire behavior and effects, and may exacerbate such effects (Donato et al. 2006; Thompson et al. 2007; Lesmeister et al. 2021; Hanson 2021; Baker and Hanson 2022). Evidence also indicates that the density and survival rates of giant sequoia regeneration are much higher in high‐severity fire areas than in lower‐severity areas (Hartesveldt et al. 1975; Harvey and Shellhammer 1991; Hanson, Chi, Baker, et al. 2024). Moreover, postfire epicormic branching, the production of new foliage from dormant buds in branches and trunks in some conifer species (Mahdizadeh and Russell 2021; Peltier et al. 2023), including giant sequoias (Stephens and Finney 2002; Meyer et al. 2024), suggests the possibility that some live sequoias may go undetected initially, prior to the growth of new crown foliage.

We investigated giant sequoia regeneration in high‐severity fire areas by returning to the Redwood Mountain Grove in Sequoia and Kings Canyon National Parks 4 years after the KNP Complex fire, following up on our investigation of giant sequoia seedling density at two years post‐fire (Hanson, Chi, Khosla, et al. 2024). Here, we focus on giant sequoia small trees (those ≥ 140 cm tall, Meyer and Safford 2011), which are “most likely to recruit into the canopy and eventually replace the parent trees” (York et al. 2013), in the two largest high‐severity fire patches, which are dominated by crown fire but are also intermixed with areas of high‐intensity surface fire.

We investigated the following null hypotheses:

Null Hypothesis 1 There is no correlation between fire severity (RdNBR values) and giant sequoia small tree density (stems/ha) in high‐severity fire areas within the two largest high‐severity fire patches in the National Park within Redwood Mountain Grove.

Null Hypothesis 2 There is no correlation between the percent of montane chaparral cover and giant sequoia small tree density in high‐severity fire areas within the two largest high‐severity fire patches in the National Park within Redwood Mountain Grove.

Null Hypothesis 3 There is no difference between the one‐year postfire data regarding distances to the nearest surviving giant sequoia and the distances to the nearest surviving giant sequoia at three years post‐fire in the National Park within Redwood Mountain Grove.

## 2 Materials and Methods

**FIGURE 1:**
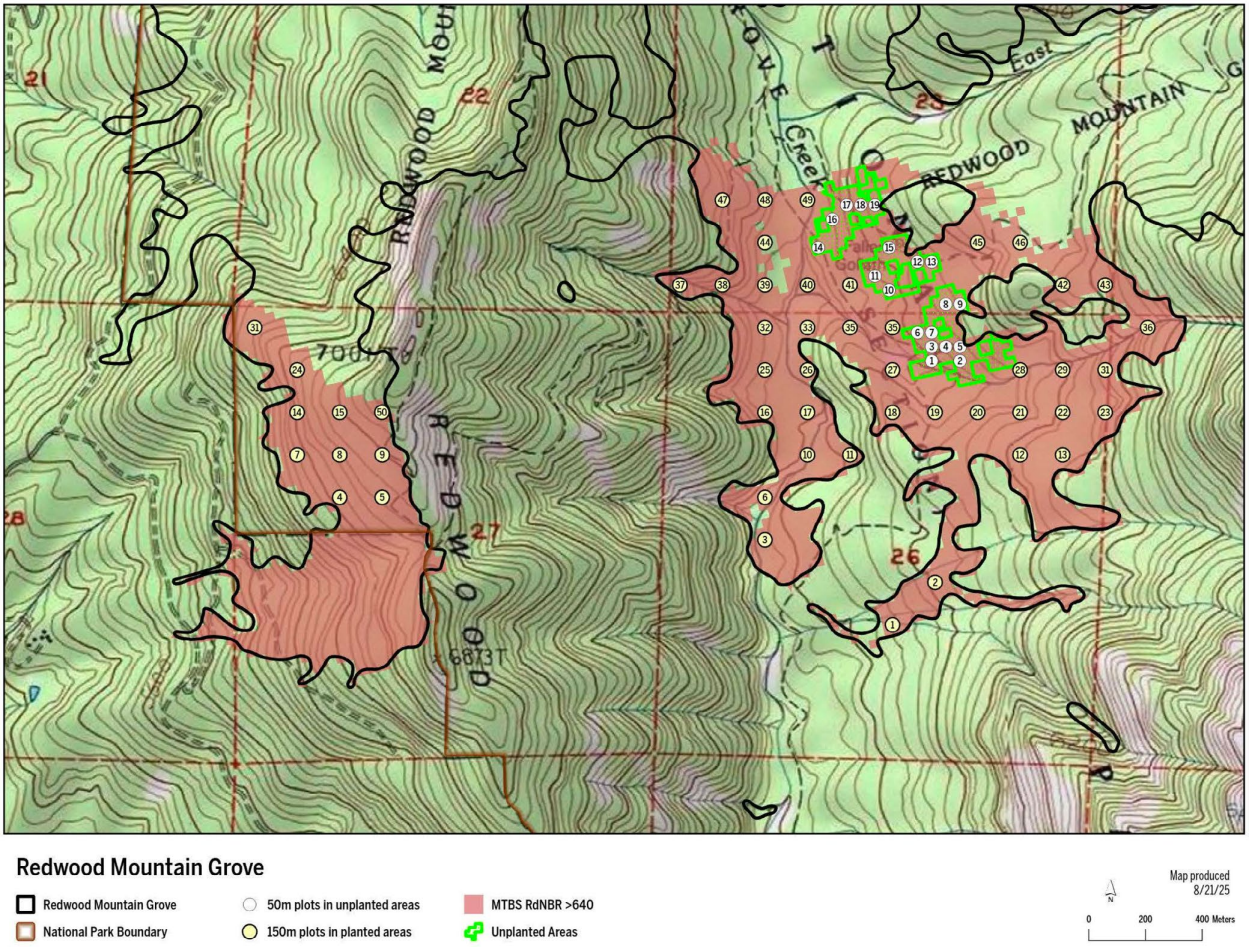
The Redwood Mountain Grove study area with fire severity and plot locations.

We investigated postfire giant sequoia reproduction in the two largest high‐severity fire patches in Sequoia and Kings Canyon National Park, within Redwood Mountain Grove, through which the KNP Complex fire burned in 2021 (Figure 1). The elevation in these two largest high‐severity fire patches ranges from 1640 to 2100 m. The mixed‐conifer forest in the study area is mainly comprised of white fir ( *Abies concolor* ), sugar pine ( *Pinus lambertiana* ), incense‐cedar ( *Calocedrus decurrens* ), giant sequoia, Jeffrey pine ( *Pinus jeffreyi* ), ponderosa pine ( *Pinus ponderosa* ), and California black oak ( *Quercus kelloggii* ), with montane chaparral mainly consisting of mountain whitethorn ( *Ceanothus cordulatus* ), deer brush ( *Ceanothus integerrimus* ), greenleaf manzanita ( *Arctostaphylos patula* ), and beaked hazelnut ( *Corylus cornuta* ).

In the fall of 2023, the Park planted sequoia and non‐sequoia conifer seedlings across a total of 200 ha of high‐severity fire areas, including most of these two large high‐severity fire patches, with the exception of three areas comprising a combined area of 13 ha that Park staff opted not to plant (Figure 1). Overall, the Park Service planted ~65,000 giant sequoia seedlings in these high‐severity fire areas (https://home.nps.gov/articles/000/restoring‐giant‐sequoia‐groves‐post‐high‐severity‐wildfires‐2024.htm). By the fall of 2024, the Park estimated that 70% of the planted seedlings had died (Brigham 2025), after which ~100/ha of the planted sequoia seedlings remained. Additional mortality of planted seedlings will likely have occurred since the fall of 2024.

All study plots were located in the two largest high‐severity fire patches, 141 and 55 ha, situated in the southern part of the Redwood Mountain Grove, within Sequoia and Kings Canyon National Parks (the southern half of the second largest high‐severity fire patch is located in Sequoia National Forest). In planted areas, a priori we established a 150‐m grid of plots across the two largest high‐severity fire patches within Sequoia and Kings Canyon National Park. In the 13 ha of unplanted areas in the largest high‐severity fire patch, a priori we established a closer, 50‐m plot grid, in order to ensure adequate sampling of the much smaller area that was not planted. All plots were located in high‐severity fire areas, with RdNBR (Relativized Differenced Normalized Burn Ratio) values > 640 (Miller and Thode 2007). We used RdNBR values from the Monitoring Trends in Burn Severity (www.mtbs.gov) satellite imagery fire severity database and our a priori plot center coordinates to determine the RdNBR value for each plot.

Similar to Stephenson, Caprio, et al. (2024), we were interested in giant sequoia old forest that had not been previously logged. Therefore, we excluded some small areas on the western flank of Redwood Mountain that were heavily logged historically and also burned at high severity in the 1987 Pierce fire and were not old forest at the time of the KNP Complex fire. In order to avoid sampling non‐grove inclusions inside the sequoia grove, we excluded plots if there were no pre‐fire live giant sequoias > 2 m in diameter at breast height (dbh) within a 100‐m radius of the plot center.

First, in a 20‐m radius around the plot center, we recorded all giant sequoia small trees (saplings ≥ 140 cm tall), and documented the ground presence or absence of sequoia cones. Second, we recorded all sequoia seedlings (< 140 cm tall) in plots with the same plot center, using a well‐established expanding plot size approach to facilitate accurate counting of sequoia seedlings, which can be extremely dense in many areas (Hanson, Chi, Khosla, et al. 2024; Meyer et al. 2024; Stephenson, Caprio, et al. 2024). If there were ≥ 2 sequoia seedlings within a 2 m radius, we used a 2‐m radius plot. If there were < 2 sequoia seedlings at a 2 m radius, but ≥ 2 at a 5 m radius, we used a 5‐m radius plot. If there were < 2 seedlings at a 5‐m radius, we used a 10 m radius, and so on up to a 20 m radius. In the sequoia seedling plots, we recorded the percent of montane chaparral cover to the nearest 5%, visually estimated. To determine whether a given sequoia seedling or small tree was within the plot, we used laser hypsometers and, in smaller‐radius plots, locking metal tape measures.

For both the planted and unplanted portions of this study, we located plot centers in the field using handheld Garmin GPS units, stopping immediately when the coordinates on the GPS unit matched the a priori coordinates of the plot center in question. To facilitate unbiased sampling, at the first moment when the two sets of coordinates matched for a given plot in this manner (at the exact moment that the distance on the GPS unit to the plot center went to zero), that precise location was used as the plot center. We gathered all field data from July 24, 2025 through August 9, 2025.

We analyzed planted and unplanted plots together, given the negligibly small number of planted seedlings present relative to total giant sequoia regeneration (planted seedlings comprised < 0.5% of the total; see Results). Moreover, our analysis of giant sequoia small trees is unlikely to be influenced at all by the planting of seedlings, since it is biologically unrealistic for seedlings (~15 cm tall) planted in October of 2023 to reach small tree stature (≥ 140 cm tall) in only one and a half growing seasons, in light of reported maximum sequoia seedling growth rates in high‐severity fire areas (Hanson, Chi, Khosla, et al. 2024).

In addition, we were interested in determining whether it is possible to accurately and completely assess the survival of giant sequoias, and therefore the distance to the nearest live, surviving giant sequoia, at only one year post‐fire in large high‐severity fire patches, in light of epicormic branching. We investigated this question by comparing one‐year postfire data from Soderberg et al. (2024) regarding the distance from plot centers to the nearest live sequoia in the large high‐severity fire patches in Redwood Mountain Grove, to three‐year postfire data from remote sensing in these same large high‐severity fire patches, regarding the distance from each plot center to the nearest live sequoias. For our three‐year postfire data, we created a 150‐m grid of plots across all areas of the two largest high‐severity fire patches in Redwood Mountain Grove. An interactive map showing the plot locations, on a 150‐m grid within the two largest high‐severity fire patches, with the 2024 NAIP imagery, can be accessed at: https://ginfo.maps.arcgis.com/apps/mapviewer/index.html?webmap=9d26797d8719408180292dd483cc72b5. We used high resolution aerial photographs from the summer of 2024, provided by the National Agriculture Imaging Program (NAIP) of the U.S. Department of Agriculture (https://naip‐usdaonline.hub.arcgis.com/), to identify live giant sequoias by visual confirmation of green foliage, within these two largest high‐severity fire patches. We then used September 2023 and October 2024 satellite imagery, and the “ruler” function from Google Earth to determine the distance from each plot center to the nearest live sequoia identified in the 2024 NAIP imagery.

We tested whether there is a correlation between RdNBR values and giant sequoia small tree density (Null Hypothesis 1) using a Spearman's rank correlation test (Glantz 2005). We also used a Spearman's rank correlation test to determine whether there is a correlation between percent montane chaparral cover and giant sequoia small tree density (Null Hypothesis 2). We used a Mann–Whitney test (Zar 2010) to determine whether there is a difference in distance (m) to the nearest live sequoia at 1 year post‐fire versus the distance (m) to the nearest live sequoia at 3 years post‐fire (Null Hypothesis 3).

## 3 Results

The mean RdNBR value in the planted areas of the large high‐severity fire patches is 978, and the mean RdNBR value in the unplanted areas of the large high‐severity fire patches is 1018. The mean giant sequoia small tree density was 75/ha (planted and unplanted plots combined). The tallest single giant sequoia small tree was 224 cm in height, at a maximum age of 3 years. The mean total giant sequoia regeneration density (seedlings plus small trees) in the 43 planted plots was 19,381/ha, and the mean total giant sequoia regeneration density in the 19 unplanted plots was 20,643/ha. Adjusted for area (unplanted areas comprised 7.7% of the total area surveyed), the overall mean total giant sequoia regeneration in the two largest high‐severity fire patches was 19,478/ha. Therefore, surviving planted seedlings, estimated to be < 100/ha, comprised less than 0.5% of the total giant sequoia regeneration at the time of our field surveys.

**FIGURE 2:**
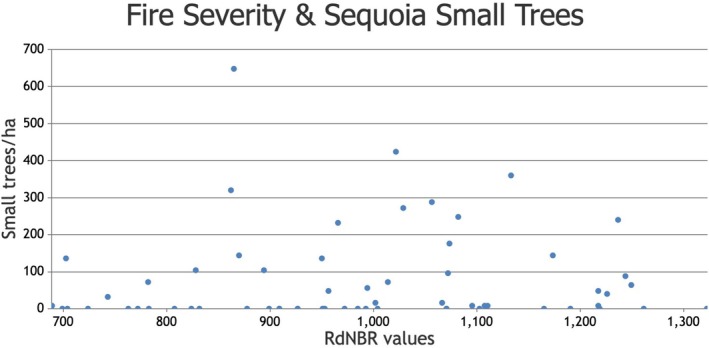
Fire severity (RdNBR values) and giant sequoia small tree density (stems/ha) in the two largest high‐severity fire patches in Redwood Mountain Grove.

**FIGURE 3:**
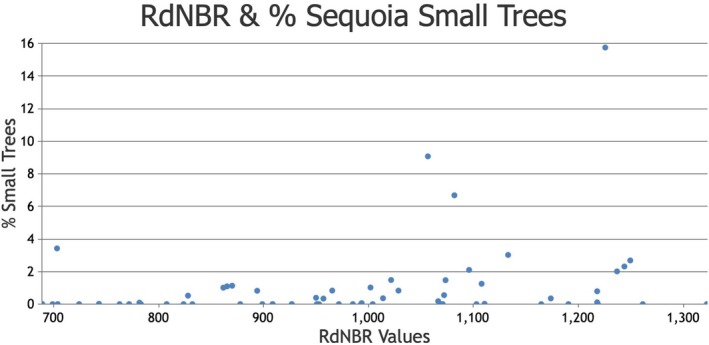
Fire severity (RdNBR values) and the percentage of all giant sequoia regeneration (seedlings and small trees combined) that is comprised of small trees.

Numerically, the density of giant sequoia small trees was more than three times higher in crown fire areas than in high‐intensity surface fire areas within the high‐severity fire category (Appendix B). However, there was no statistically significant correlation between fire severity and giant sequoia small tree density (*r*_s_ = 0.166, *p* = 0.197, *n* = 62, Figure 2 and Appendix A). In an a posteriori Spearman's rank correlation analysis of our 62 field plots, we found that the percentage of all giant sequoia regeneration that is comprised of small trees (i.e., small trees per ha divided by all regeneration per ha, converted to percent) increases significantly with higher RdNBR values within the high‐severity fire category (*r*_s_ = 0.300, *p* = 0.018, *n* = 62, Figure 3 and Appendix B). The highest proportions of giant sequoia small trees were in crown fire areas, in particular those with RdNBR values > 1000 (Figure 3).

**FIGURE 4:**
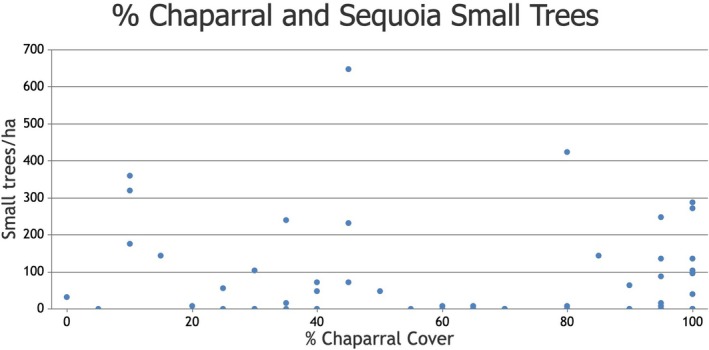
Percent montane chaparral cover and giant sequoia small tree density (stems/ha) in the two largest high‐severity fire patches in Redwood Mountain Grove.

There was no correlation between percent montane chaparral cover and giant sequoia small tree density (*r*_s_ = −0.107, *p* = 0.408, *n* = 62, Figure 4). Mean montane chaparral cover was 64.7%, and ranged from 0% to 100%.

The distances to live, surviving giant sequoias in the two largest high‐severity fire patches in Redwood Mountain Grove at one year post‐fire were significantly larger (mean = 258 m, *n* = 46) than the distance we recorded at three years post‐fire (mean = 158 m, *n* = 58) based on high resolution aerial photographs and satellite imagery, and null hypothesis 3 was rejected (U = 924.5, *p* = 0.007, Appendix C). In 98% of plots, distances to live sequoias were less than the ~500‐m seed dispersal distance by wind for giant sequoias.

We found giant sequoia regeneration in 100% of our field plots, and giant sequoia cones were present, typically in high abundance, in 100% of our plots.

## 4 Discussion

**FIGURE 5:**
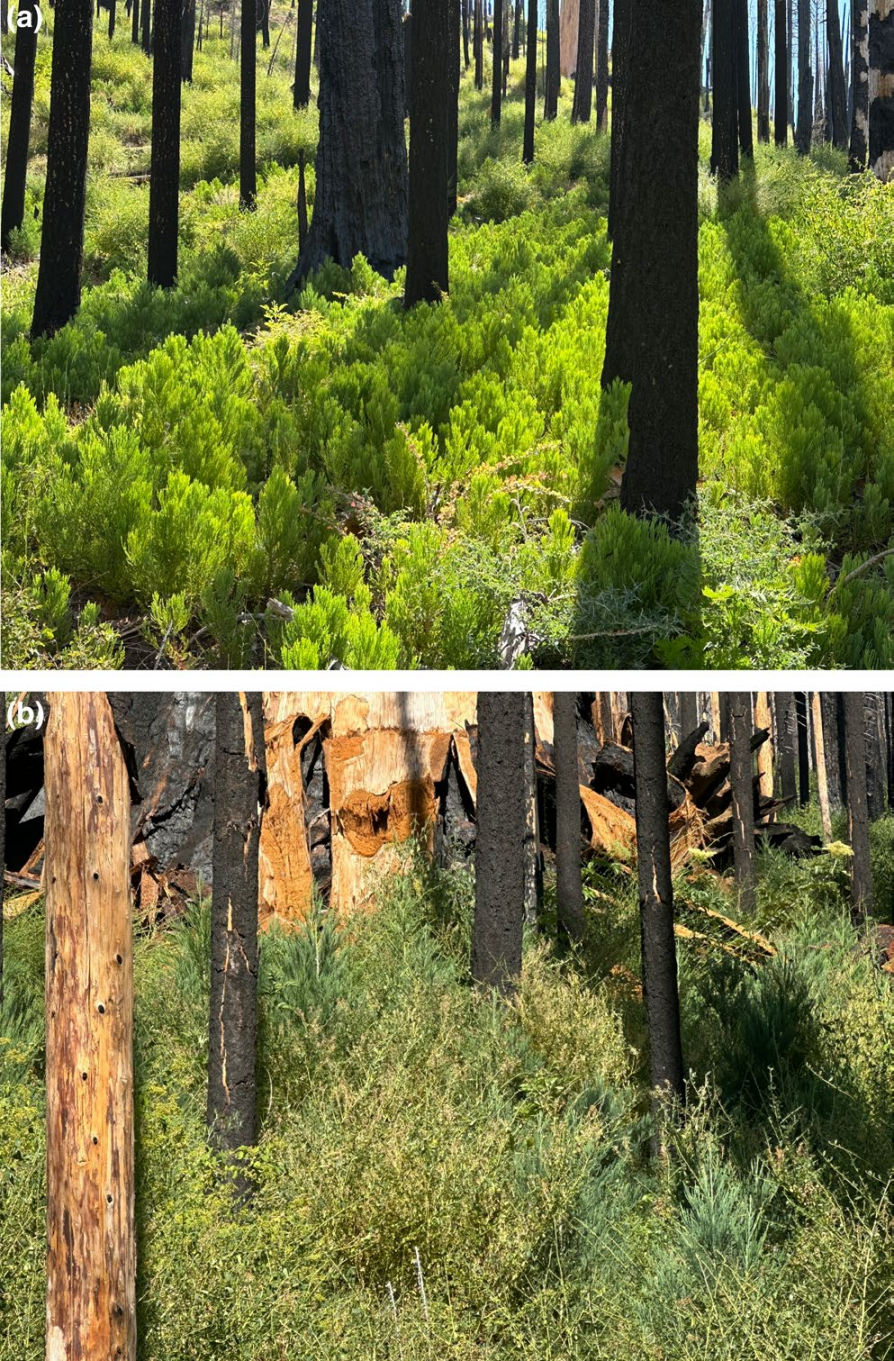
Abundant giant sequoia regeneration in Redwood Mountain Grove (a) deep in the interior of a large crown fire area, and (b) in a large crown fire area, with many giant sequoia small trees overtopping nearly 100% montane chaparral cover.

We found abundant, rapidly growing giant sequoia regeneration in the two largest high‐severity fire patches in the Redwood Mountain Grove (Figure 5). We found no relationship between fire severity and giant sequoia small tree regeneration density within these patches, and found no relationship between montane chaparral cover and giant sequoia small tree regeneration density. Sequoia regeneration and sequoia cones were present in 100% of our 62 20‐m‐radius field plots. Tree planting in portions of the large high‐severity fire patches did not appear to influence our conclusions. Planted seedlings comprised less than one‐half of 1% of giant sequoia regeneration in areas where planting occurred.

Further, we found much shorter distances to the nearest live giant sequoia within these two largest high‐severity fire patches, compared to data reported from one year post‐fire in the same patches. This suggests that some live sequoias were missed initially, possibly due to the lack of easily visible remaining live foliage from a ground‐level view at one year post‐fire and prior to the production of new green foliage through epicormic branching.

Our a posteriori analysis of the relationship between RdNBR values and the percentage of all sequoia regeneration that is comprised of small trees revealed a significant positive correlation, with the highest percentages in the crown fire areas with RdNBR values > 1000. Our interpretation of this finding is that, in the crown fire portions of larger high‐severity fire patches, giant sequoia seedlings have the best chance of surviving to become small trees. Two possible explanations for this result occur to us, both of which warrant investigation in future studies. First, crown fire areas would likely result in the consumption of all pre‐fire duff and litter on the forest floor, perhaps more so than high‐intensity surface fire areas. Second, in crown fire areas, the foliage of mature sequoias and other conifers is consumed, unlike high‐intensity surface fire areas where the foliage is killed by radiant heat. The possible additional consumption of duff and litter, combined with the consumption of the abundant, dense foliage atop large trees, would likely deposit additional nutrient‐rich ash on the forest floor, which could aid growth and survival of sequoia seedlings and small trees.

Our finding, that there is no correlation between percent montane chaparral cover and giant sequoia small tree density, also warrants further future investigation. Previous research has found that different conifer species can have positive or negative correlations with percent montane chaparral cover (Shatford et al. 2007). It is still unclear whether postfire giant sequoia regeneration has no relationship with percent montane chaparral cover, as we found here, or a positive relationship, as found in Hanson, Chi, Baker, et al. (2024) at 6 years after high‐severity fire.

**FIGURE 6:**
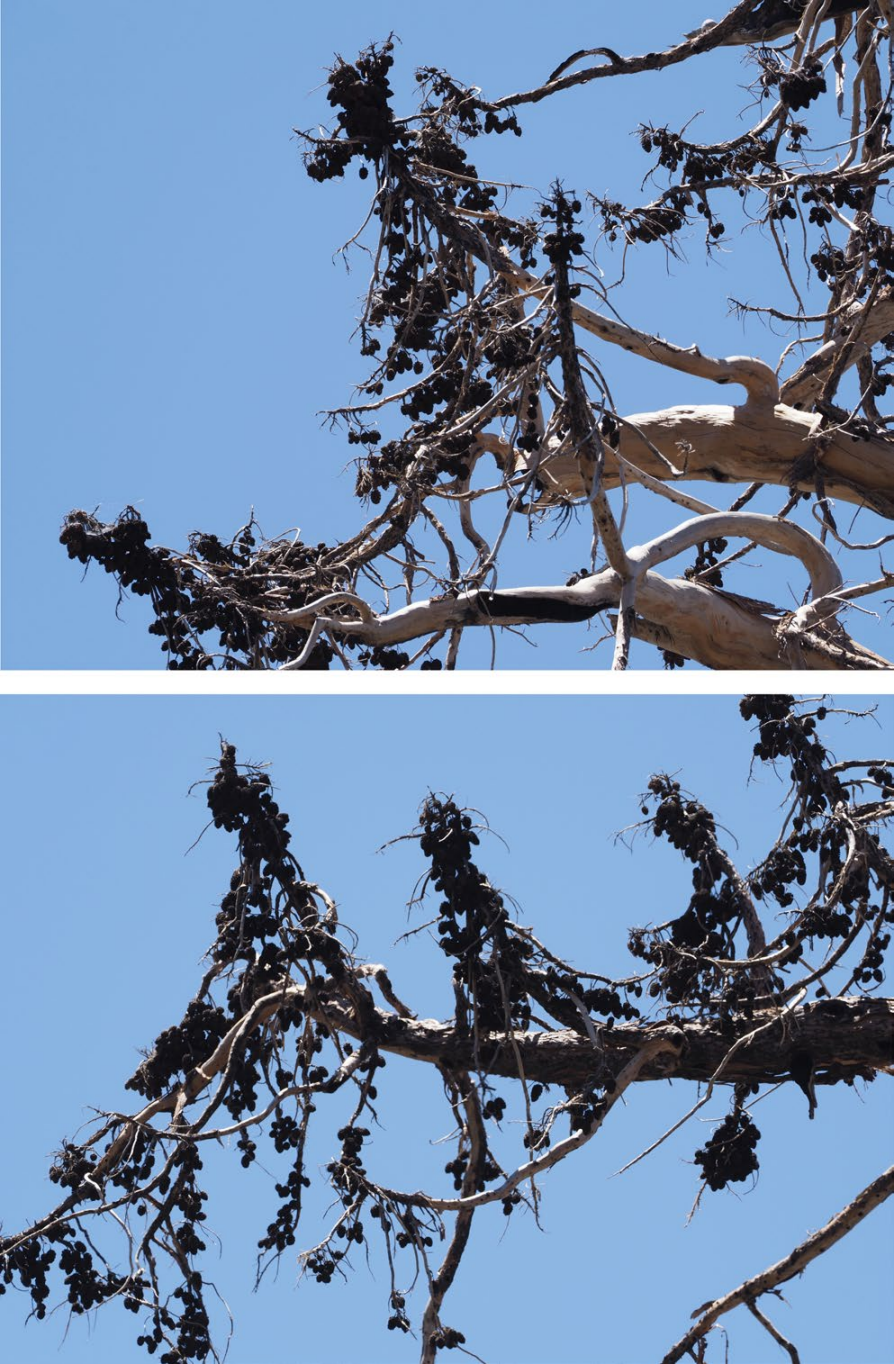
Typical dense clusters of cones tightly packed on branches of large giant sequoia snags in crown fire areas at 4 years post‐fire in Redwood Mountain Grove.

A giant sequoia regeneration density of 10,946/ha was reported at year 1 post‐fire in the large high‐severity fire patches in Redwood Mountain Grove (Soderberg et al. 2024). Initial modeling projected a 91.7% reduction in giant sequoia regeneration density in these areas from year 1 post‐fire to year 4 post‐fire, based on data from lower‐severity prescribed fires (Table 1 of Stephenson, Caprio, et al. 2024). This would equate to a giant sequoia regeneration density of ~909/ha by year 4 post‐fire in these high‐severity fire patches within Redwood Mountain Grove. However, we found a mean sequoia regeneration density of 19,478/ha at year 4 post‐fire in these large high‐severity fire patches, more than 21 times denser than initially projected. We believe the main reason that our results differ from previous projections is that those projections (Soderberg et al. 2024; Stephenson, Caprio, et al. 2024) were based on giant sequoia regeneration and seedling mortality in different conditions, specifically lower‐severity prescribed fires. Previous research indicates that giant sequoia regeneration has far higher survival in high‐severity areas than in lower‐severity sites (Hartesveldt et al. 1975; Harvey and Shellhammer 1991). Similarly, giant sequoia regeneration growth rates are also far higher in high‐severity patches than in lower‐severity fire areas. This is likely due in large part to the greatly increased sunlight in larger high‐severity fire patches compared to lower‐severity fire areas (Meyer and Safford 2011; Hanson, Chi, Khosla, et al. 2024; Hanson, Chi, Baker, et al. 2024), and increased production of nutrient‐rich mineral ash from high to complete consumption of the duff and litter on the forest floor in high‐severity areas (Hanson, Chi, Khosla, et al. 2024; Hanson, Chi, Baker, et al. 2024). In addition, recent projections regarding a lack of giant sequoia regeneration in high‐severity fire patches were based on the hypothesis that sequoia cones and seeds are consumed in crown fire areas, killing the seeds (Soderberg et al. 2024; Stephenson, Caprio, et al. 2024; Stephenson, Soderberg, et al. 2024; Keeley and Pausas 2025). We found giant sequoia cones in all crown fire areas, and dense clusters of cones still cling to the branches of giant sequoia snags at four years post‐fire, where crown fire occurred (Figure 6). Our results do not indicate that giant sequoias are maladapted to crown fire (Stephenson, Soderberg, et al. 2024; Keeley and Pausas 2025) and provide further support for the longstanding characterization of giant sequoias as serotinous (Harvey et al. 1980; Weatherspoon 1990; Harvey and Shellhammer 1991).

Our results indicate successful giant sequoia regeneration in the high‐severity and crown fire areas of Redwood Mountain Grove. Of course, there are notable variables and differences among unique giant sequoia grove locations. Soils may differ within and between groves. Some groves are on very steep and rocky terrain. Others are at or near the edge of the range of giant sequoias. This suggests to us that attempts to apply a singular seedling density threshold or objective across the entire range of the species are unwarranted.

Some previous studies have articulated the hypothesis that any portion of a grove with seedling densities below a certain numerical threshold will be “lost” as part of the grove due to total regeneration failure (Meyer et al. 2024; Soderberg et al. 2024; Stephenson, Caprio, et al. 2024; Stephenson, Soderberg, et al. 2024; Keeley and Pausas 2025). However, previous research has not investigated the question of whether there is any lower sequoia regeneration density threshold, below which all sequoia regeneration will die, causing that portion of the grove to be lost, and Sequoia and Kings Canyon National Parks indicate that there is no such threshold (USDOI 2023).

In addition, as discussed above, giant sequoia regeneration survives at vastly higher rates in high‐severity fire areas than in surface fire areas. Historical densities of large (≥ 107 cm dbh; Harvey et al. 1980) giant sequoias ranged from 3.1 to 6.9/ha, based on extensive surveys conducted in the mid‐1930s in unlogged giant sequoia groves (Been 1938). If a given crown fire area has, for example, a sequoia regeneration density of 100/ha or 10/ha in the early post‐fire years, and the sequoia seedlings and small trees are growing vigorously and surviving at high rates, such areas could easily meet or exceed the natural density range of large sequoias decades later when the stand reaches maturity. Furthermore, the distance to live sequoias in the largest high‐severity fire patches in Redwood Mountain Grove is much shorter than initially believed, and the wind dispersal distance for giant sequoia seeds is > 500 m (Harvey et al. 1980). Moreover, when high‐severity fire areas reburn in a subsequent fire, they experience mostly lower‐severity surface fire (Coppoletta et al. 2016). Even small, young sequoia trees tend to survive lower‐severity fires (Stephens and Finney 2002). Moving forward, it will be important to monitor high‐severity fire trends and giant sequoia regeneration, especially in light of potential impacts of climate change and drought cycles (Enright et al. 2015).

With regard to management implications, our results suggest that land managers may have considerably more flexibility than previously believed (USDOI 2023; Meyer et al. 2024; Soderberg et al. 2024; Stephenson, Caprio, et al. 2024; Stephenson, Soderberg, et al. 2024; Keeley and Pausas 2025) to allow and embrace the occurrence of mixed‐severity fires in giant sequoia groves, as well as the natural succession processes following such events (though more research is needed, with more time since fire, on additional groves to confirm this). This is good news for giant sequoia conservation, given that such a management approach would avoid the adverse impacts that are often associated with mechanical thinning and postfire logging (Donato et al. 2006; Thompson et al. 2007; Lesmeister et al. 2021; Baker and Hanson 2022; Hanson, Chi, Baker, et al. 2024).

## Author Contributions

**Chad T. Hanson:** conceptualization (lead), data curation (lead), formal analysis (lead), funding acquisition (lead), investigation (equal), methodology (lead), project administration (lead), resources (equal), software (lead), supervision (lead), validation (equal), visualization (lead), writing – original draft (lead), writing – review and editing (equal). **Tonja Y. Chi:** conceptualization (supporting), data curation (equal), formal analysis (supporting), funding acquisition (supporting), investigation (equal), methodology (equal), project administration (supporting), resources (supporting), software (supporting), supervision (supporting), validation (equal), visualization (supporting), writing – original draft (supporting), writing – review and editing (equal). **Maya Khosla:** conceptualization (supporting), data curation (equal), formal analysis (supporting), funding acquisition (supporting), investigation (equal), methodology (equal), project administration (supporting), resources (supporting), software (supporting), supervision (supporting), validation (equal), visualization (supporting), writing – original draft (supporting), writing – review and editing (equal). **Craig Swolgaard:** conceptualization (supporting), data curation (equal), formal analysis (supporting), funding acquisition (supporting), investigation (equal), methodology (equal), project administration (supporting), resources (supporting), software (supporting), supervision (supporting), validation (equal), visualization (supporting), writing – original draft (supporting), writing – review and editing (equal).

## Funding

This work was supported by Environment Now foundation, 2025.

## Conflicts of Interest

The authors declare no conflicts of interest.

## Supporting information


**Appendix A.** Raw field plot data and plot center coordinates, Redwood Mountain Grove.

**Appendix B.** Spreadsheet for RdNBR values and percent giant sequoia small trees.

**Appendix C.** Plot coordinates and distance to the nearest live giant sequoias from plot centers.

## Data Availability

All the required data are uploaded as Appendices A through C.
